# *Paracrias pluteus* (Hymenoptera, Eulophidae) in Brazil: new distribution and host records, and with a new host group for *Paracrias*

**DOI:** 10.3897/zookeys.102.1343

**Published:** 2011-06-02

**Authors:** Tiago G. Pikart, Gabriely K. Souza, Valmir A. Costa, Christer Hansson, José C. Zanuncio

**Affiliations:** 1 Departamento de Biologia Animal, Universidade Federal de Viçosa, 36570-000, Viçosa, Minas Gerais, Brazil; 2 AInstituto Biológico/APTA, 13012-970, Campinas, São Paulo, Brazil, C.P. 70; 3 The Natural History Museum, London, England

**Keywords:** Bruchinae, seeds, parasitoid, Hymenoptera, Eulophidae, *Paracrias pluteus*, new distribution, Brazil

## Abstract

Bruchines damage agricultural crops and trees, reducing the quantity and quality of the seeds. The aim of this study is to record, for the first time, *Paracrias pluteus* as a parasitoid on the immature stages of *Sennius spodiogaster* and *Sennius cupreatus* on seeds of *Melanoxylon brauna* in Teixeiras, Minas Gerais State, Brazil. *Paracrias pluteus* is a parasitoid without previous host records and known only from Costa Rica. Specimens obtained in this study add to knowledge of the biology of *Paracrias* species with a new host group (Chrysomelidae: Bruchinae), and the first host record and a new distribution for *Paracrias pluteus*.

## Introduction

Bruchines (Coleoptera: Chrysomelidae: Bruchinae) are considered pests of seeds of native and cultivated legumes in Latin America, causing damage to several economically important agricultural species (Rojas-Rousse et al. 2007) such as *Glycine max* (Costa et al. 2007), *Phaseolus coccineus*, *Phaseolus vulgaris*, *Phaseolus lunatus* (Hansson et al. 2004, Bonet 2008), *Vigna radiata* (Somta et al. 2008), *Vigna unguiculata* (Aebi et al. 2008), and tree species such as *Enterolobium contortisiliquum* (Morandini and De Viana 2009), *Melanoxylon brauna* (Santos et al. 1991), *Mimosa bimucronata* (Silva et al. 2007), *Sclerolobium* sp. (Santos et al. 1997) and *Senna multijuga* (Sari and Ribeiro-Costa 2005).

Infestations of bruchines result in a large reduction of the quantity and quality of seeds, making them unsuitable for human consumption and for agricultural use (Somta et al. 2008). Currently the most efficient method to control these infestations on a large scale is to fumigate the seeds with chemicals (Sing et al. 2008), but this method has economic, social and environmental implications (Somta et al. 2008). Therefore, control measures including plant resistance (Ignacimuthu et al. 2000, Schmale et al. 2003, Appleby et al. 2004), plant extracts with biocide activity (Raja et al. 2004, Koona et al. 2005), and natural enemies (Sanon et al. 1998, Gauthier et al. 1999, Schmale et al. 2006) such as parasitoids of the families Braconidae, Eulophidae, Pteromalidae (Schmale et al. 2001, 2002, Rojas-Rousse et al. 2007) and Trichogrammatidae (Pintureau et al. 1999) constitute more sustainable alternatives.

The aim of this study is to record, for the first time, the occurrence of *Paracrias pluteus* Hansson, 2002 (Hymenoptera: Eulophidae) as a parasitoid on immature stages of *Sennius* spp. (Coleoptera: Chrysomelidae: Bruchinae) on *Melanoxylon brauna* Schott in Teixeiras, Minas Gerais State, Brazil.

## Materials and methods

Seeds of *Melanoxylon brauna* infested by bruchines were studied in the Laboratório de Sementes Florestais (LASF), Universidade Federal de Viçosa (UFV) in Viçosa (20°46'11"S, 42°52'31"W), Minas Gerais State, Brazil. Seeds were collected in Teixeiras, Minas Gerais State, in September 2009 and sent to LASF where they were stored in plastic bags in a room of the laboratory without temperature, humidity or photoperiod control. Insects that emerged from the seeds were collected and stored in 70% alcohol for subsequent identification. Two bruchine species were and identified as *Sennius spodiogaster* Kingsolver, 1987 and *Sennius cupreatus* Kingsolver, 1987 (Coleoptera: Chrysomelidae: Bruchinae). Apart from the bruchines, three females and nine males of a parasitoid species emerged. This parasitoid was subsequently identified as *Paracrias pluteus*.

## Results and discussion

*Paracrias pluteus* is a parasitoid without previous host records and known only from Costa Rica (Hansson 2002). The knowledge of the biology of *Paracrias* is poor, the only known hosts are Curculionidae beetles that attack seeds (Schauff 1985) or buds (Woolley and Schauff 1987). The specimens obtained in this study add to this knowledge with a new host group (Chrysomelidae: Bruchinae) for the genus, and the first host record for *Paracrias pluteus*.

*Paracrias* species occur exclusively in the New World with the greatest diversity in the tropics (Hansson 2002). In 2001 eight species were known, but Gumovsky (2001) and Hansson (2002) increased this number to 65. Hansson (2009) released an interactive identification key for *Paracrias* species online (http://www.neotropicaleulophidae.com/Index.html), but species described by Gumovsky were not included because their descriptions did not include most of the characters used in the key.

Even though the number of described species of *Paracrias* is relatively high, *Paracrias pluteus* is only the fifth species of the genus known to occur in Brazil. *Paracrias* was described by Ashmead (1904) from specimens collected in Brazil, with the singular included species *Paracrias laticeps* Ashmead. The other species that occur in Brazil are *Paracrias panamensis* Gumovsky (Gumovsky 2001), *Paracrias beus* Schauff (De Santis and Fidalgo 1994) and *Paracrias petilicornis* Hansson (Hansson 2002).

**Figure 1.**
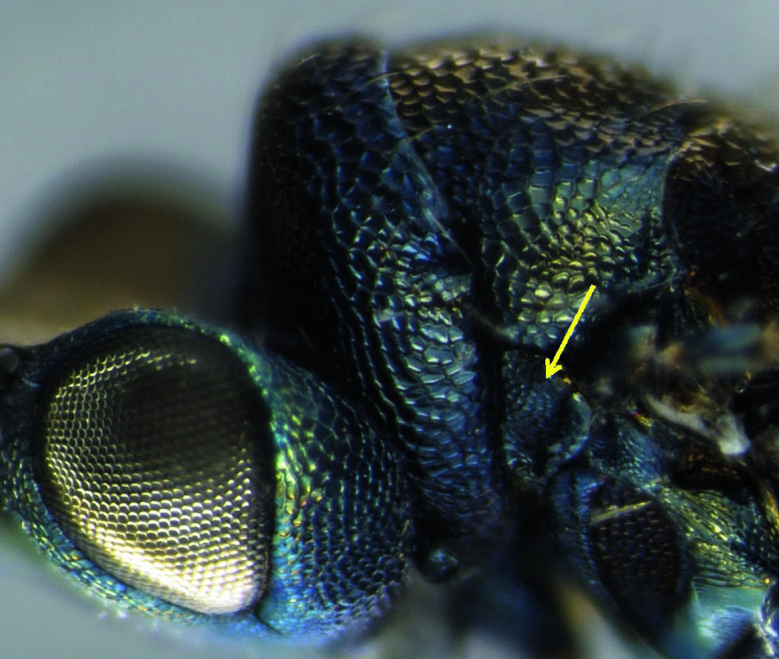
Lateral view of *Paracrias pluteus* adult female. Lateral view of *Paracrias pluteus *Hansson, 2002 (Hymenoptera: Eulophidae) adult female with detail to prepectus entirely reticulated and its less bright body color. Teixeiras, Minas Gerais State, Brazil.

**Figure 2.**
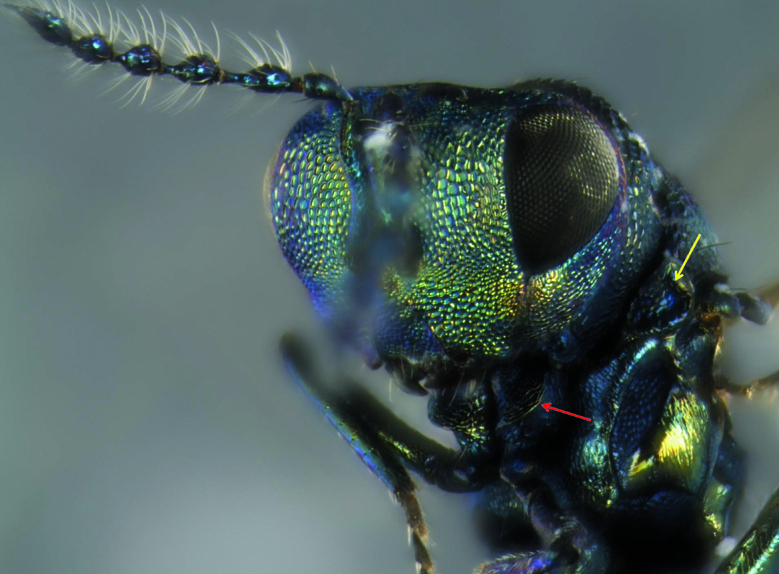
Frontal view of *Paracrias pluteus* adult male head. Frontal view of *Paracrias pluteus* Hansson, 2002 (Hymenoptera: Eulophidae) adult male head with detail to prepectus entirely reticulated (yellow arrow), procoxae carina (red arrow), its antennae and its bright body color. Teixeiras, Minas Gerais State, Brazil.

*Paracrias pluteus* belongs to the *ordinatus *species-group, which is characterized by the forewing which has a narrow membrane along the fore margin of marginal vein, post-marginal vein absent, a very large speculum, and wing membrane distal to speculum sparsely setose (Hansson 2002). Within this group, *Paracrias pluteus* is distinguished mainly by having a strong, transverse and flat carina on procoxae and with prepectus fully reticulated (Figs 1 and 2). Males are distinguished from females by having all flagellomeres distinctly separated, a longer petiole and by being more colorful (Figs 1 and 2).

*Melanoxylon brauna* is a plant of high economic value, and *Sennius spodiogaster* and *Sennius cupreatus* may destroy as much as 50% of its seeds (Santos et al. 1991). Studies on the biology of *Paracrias pluteus* may provide important information for its use in programs of biological control of these bruchines.
